# Germinated brown rice (GBR) reduces the incidence of aberrant crypt foci with the involvement of β-catenin and COX-2 in azoxymethane-induced colon cancer in rats

**DOI:** 10.1186/1475-2891-9-16

**Published:** 2010-03-26

**Authors:** Saiful Yazan Latifah, Nurdin Armania, Tan Hern Tze, Yaacob Azhar, Abdul Hadi Nordiana, Saad Norazalina, Ithnin Hairuszah, Moin Saidi, Ismail Maznah

**Affiliations:** 1 Department of Biomedical Science, Faculty of Medicine and Health Sciences, Universiti Putra Malaysia, 43400 UPM Serdang, Selangor Malaysia; 2 Department of Nutrition and Dietetics, Faculty of Medicine and Health Sciences, Universiti Putra Malaysia, 43400 UPM Serdang, Selangor Malaysia; 3 Department of Pathology, Faculty of Medicine and Health Sciences, Universiti Putra Malaysia, 43400 UPM Serdang, Selangor Malaysia; 4 Laboratory of Molecular Biomedicine, Institutes of Bioscience, Universiti Putra Malaysia, 43400 UPM Serdang, Selangor Malaysia

## Abstract

Chemoprevention has become an important area in cancer research due to the failure of current therapeutic modalities. Epidemiological and preclinical studies have demonstrated that nutrition plays a vital role in the etiology of cancer. This study was conducted to determine the chemopreventive effects of germinated brown rice (GBR) in rats induced with colon cancer. GBR is brown rice that has been claimed to be richer in nutrients compared to the common white rice. The male *Sprague Dawley *rats (6 weeks of age) were randomly divided into 5 groups: (G1) positive control (with colon cancer, unfed with GBR), (G2) fed with 2.5 g/kg of GBR (GBR (g)/weight of rat (kg)), (G3) fed with 5 g/kg of GBR, (G4) fed with 10 g/kg of GBR and (G5) negative control (without colon cancer, unfed with GBR). GBR was administered orally once daily via gavage after injection of 15 mg/kg of body weight of azoxymethane (AOM) once a week for two weeks, intraperitonially. After 8 weeks of treatment, animals were sacrificed and colons were removed. Colonic aberrant crypt foci (ACF) were evaluated histopathologically. Total number of ACF and AC, and multicrypt of ACF, and the expression of β-catenin and COX-2 reduced significantly (*p *< 0.05) in all the groups treated with GBR (G2, G3 and G4) compared to the control group (G1). Spearman rank correlation test showed significant positive linear relationship between total β-catenin and COX-2 score (Spearman's rho = 0.616, *p *= 0.0001). It is demonstrated that GBR inhibits the development of total number of ACF and AC, and multicrypt of ACF, reduces the expression of β-catenin and COX-2, and thus can be a promising dietary supplement in prevention of colon cancer.

## Background

Colon cancer develops through a multistage process that could be recognized at a histopathological level by progression from mucosa to invasive carcinoma. Colorectal cancer is becoming increasingly common in Asian countries while remains the second leading cause of cancer death in the United States. Approximately 110,000 new cases are diagnosed each year [1]. Epidemiological data suggest that diet is a major factor in the etiology of colon cancer [2].

GBR is brown rice, which has been soaked in water for up to a day and has a germ of approximately 1 mm long. During germination, nutrients in the brown rice change drastically. Nutrients that increase in content include γ-amirobutyric acid (GABA), dietary fiber, inositols, ferulic acid, phytic acid, tocotrienols, magnesium, potassium, zinc, γ-oryzanol, and prolylendopeptidase inhibitor [3]. According to Ken'ichi, germinated brown rice contained more total ferulic acid (126%), total dietary fiber (145%), soluble dietary fiber (120%) and insoluble dietary fiber (150%) compared to the brown rice [4]. Aberrant crypt foci (ACF) are a cluster of colorectal crypts with abnormal morphology that was first discovered in mice treated with azoxymethane [5]. In 1991, Prestlow has proposed that aberrant crypt foci in methylene blue-stained of colon as putative preneoplastic lesions that could be found in rodents as well as in human [6]. ACF were identified in rat colon, appearing a few weeks after treatment with carcinogen and becoming larger with time, with more marked nuclear atypia or dysplasia [7]. The aberrant crypts stained more darkly, were larger and had a thicker epithelial lining and a larger pericryptal zone than normal crypts [8]. Further investigation had established ACF as biomarkers of cancer risk in azoxymethane (AOM)-treated in rodents [9].

β-catenin, which was originally a cadherin-binding protein, has recently been proven to function as a transcriptional activator when it formed a complex with members of the T cell factor (Tcf) family of DNA binding protein [10,11]. Activation of the β-catenin-Tcf pathway results in the accumulation of β-catenin in the cytosol and nucleus [11,12]. Target genes of the β-catenin-Tcf pathway were determined to be growth-promoting genes, which suggest that β-catenin-Tcf is an oncogenic pathway. It is also known that β-catenin levels are regulated by the degradation of the protein through the ubiquitin-proteasome pathway [13]. Mutation in the *Adenomatous polyposis coli *(*APC*) or *β-catenin *genes, are associated with majority of human and rat colon tumors [13,14] and were proved to repress the degradation of the protein and generate β-catenin accumulations [15], which leads to activation of the oncogenic β-catenin-Tcf pathway. Excessive β-catenin protein has been shown in many colon cancers of rats and human [13]. Yamada *et al*. [16] had demonstrated that *β-catenin *gene mutations and accumulations of the protein are involved in the initial stage of colon carcinogenesis induced in rats by AOM.

Cyclooxygenase (COX) catalyses the rate limiting step in the conversion of arachidonic acid to prostaglandin H2 (PGH2), which is further metabolized to form PGD2, PGE2, PGF2α, PGI2, thromboxanes A2 and leukotrienes [17-19]. COX exists in at least two different isoforms COX-1 and COX-2, which have a high degree of structural and enzymatic homology but pharmacologically distinct [20]. COX-2 is an inducible enzyme produced mainly in inflammatory cells, inflammatory sites and colorectal carcinomas [19,21]. β-catenin-Lef (lymphoid enhancer-binding factor) complex acts as a crucial regulators of COX-2 mRNA, protein and PGE2 expression [22]. It has been demonstrated that the COX-2 5' regulatory region contains the consensus Lef-1 binding motive, thus supporting the hypothesis that COX-2 expression is mediated through Wnt (the name derived from lnt-1 and Drosophila wingless) signalling pathway. In animal studies, COX-2 mRNA and protein level in adenomatous tissue is three fold higher than normal mucosa of the same Min mouse bearing a mutation in the *APC *gene and markedly elevated in carcinogen-induced rat colonic tumours [23,24].

Studies have led to the recognition of the importance of COX-2 in colorectal tumorigenesis and in the development of intestinal neoplasms in genetically manipulated animal models, in which overexpression of COX-2 in colon cancer as well as its established cell lines is frequently demonstrated [25]. Tsujii *et al*. has also demonstrated that COX-2 overexpression enhances neovascularization thus conferring survival advantage of colon cancer cells [26].

Continuous intake of germinated brown rice has been predicted to prevent cancer of colon, but there is no scientific proof on that, as yet [27]. The goal of this study was to determine the chemopreventive properties of germinated brown rice in rat induced with colon cancer.

## Materials and methods

### Animals, diets, carcinogen and GBR

Five-week-old male *Sprague Dawley *rats were purchased from the Faculty of Veterinary Medicine, UPM. Animal Care and Use Ethic was obtained from the Animal Care and Use Committee (ACUC), Faculty of Medicine and Health Sciences, Universiti Putra Malaysia (ACUC No; UPM/FPSKPADS/FO1-00172). AOM was purchased from Sigma Chemical Company, St. Louis USA. The rats were housed in plastic cages (2 rats per cage) with woodchip bedding in a well ventilated room at room temperature, 29-32°C; 50-60% relative humidity; 12 hours light and darkness cycle. Hygienic conditions were maintained by weekly changes of the woodchip beds. GBR was kindly supplied by the Laboratory of Molecular Biomedicine, Institute of Bioscience, Universiti Putra Malaysia. Briefly, GBR was soaked at 32°C for 24 hours until germinated. Subsequently, GBR was ground using Rotary type grinding and pulverizing machines (RT-34) to produce powder. The GBR powder was dissolved in distilled water according to the dosage/treatment.

### Experimental procedures

Following a one week of acclimatization, the male *Sprague Dawley *rats were divided into five groups (n = 10), which were (G1) positive control (with colon cancer, unfed with GBR), (G2) fed with 2.5 g/kg of GBR (GBR (g)/weight of rat (kg), (G3) fed with 5 g/kg of GBR, (G4) fed with 10 g/kg of GBR and (G5) negative control (without colon cancer, unfed with GBR). At 6 weeks of age, the animals in groups 1-4 were injected with AOM intraperitonially (i.p) (15 mg/kg body weight) once weekly for two weeks. At 9 weeks of age, rats in group 2-4 were treated with GBR at 2.5 g/kg, 5 g/kg and 10 g/kg (weight of GBR (g)/weight of rat (kg)) by oral administration via gavage once daily for 8 weeks. The animals were fed with commercial basal diet in the form of pellet throughout the experimental period. The GBR free animals were given distilled water (that has been used to dissolve GBR prior to treatment) via gavage throughout the experimental period. Body weight of the rats was recorded weekly until the termination of the study. Upon termination, all animal were sacrificed by decapitation under chloroform anesthesia. Colon was removed and cut longitudinally. The colon was washed with phosphate-buffered saline, fixed in 10% of formalin and stained with hematoxylin and eosin. The total number of ACF and AC per colon was recorded.

### Immunohistochemical analysis of β-catenin and COX-2

The paraffin embedded sections were heated at 60°C for 60 min, deparaffinized in xylene, and rehydrated through graded alcohol at room temperature. Four-μm-thick section obtained from whole part of the colon of each rat was treated with 3% of bovine serum albumin (BSA) for one hour at room temperature to block the non-specific antigen sites, and subsequently incubated with primary antibody for another one hour. The expression of β-catenin was determined using a monoclonal antibody raised against a peptide corresponding to the C-terminus of mouse β-catenin (Transduction Laboratories, Lexington, KY) (1:200). COX-2 protein expression was detected by tissue culture supernatant of rabbit IgG (Neomarkers, Fremont, CA) raised against a synthetic peptide from C-terminus of rat COX-2 (1:100). All the antibodies were diluted in antibody diluent (LabVision, UK). Streptavidin-biotin complex peroxidase kit (Code No. K0690, LSAB^®^+ Kit, DAKO, USA) was used as the secondary antibody. The peroxidase activity was developed by incubating the slides in the substrate 3,3'-diaminobenzidine (DAB) (LabVision, UK). A semi-quantitative scoring system adopted from Rajnakova *et al*. [28] was used to score the staining by antibody against β-catenin and COX-2. The immunoreactive score was determined by summing up the extent and intensity of the staining (score = extent+intensity) of five fields, randomly selected from each section of colon per rat. The extent of positive cells was evaluated using the following scale: 0, no staining of the mucosal epithelial cells in any field; 1+, <25% of the epithelium stain positive; 2+, 25-50% stain positive; 3+, 50-75% stain positive; and 4+, >75% stain positive. As for the strength of intensity of staining, evaluation was based on the following scale; 0, no staining of the epithelial cells; 1+, mild staining; 2+, moderate staining; and 3+, intense staining. The maximum score after summation was 7 and the minimum was 0.

### Statistical analysis

Difference in ACF and AC incidences among rats fed with GBR was compared by One Way ANOVA, and difference in body weight was analyzed by Two Way ANOVA. Duncan method for multiple comparisons was used to determine the significance among each group. Non-parametric Kruskal-Wallis test and Mann-Whitney U-test were used to compare the expression of β-catenin and COX-2. Correlation between the two proteins was analyzed by Spearman rank correlation test. The mean score was calculated by summation of the extent and intensity of the staining (score = extent+intensity) of five field, randomly selected from each section of colon/rat. The data were presented as mean ± SEM.

## Results

**Figure 1:**
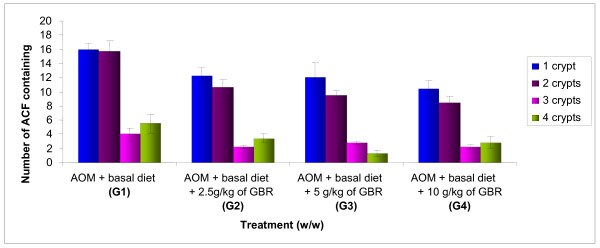
**Effect of different percentage of germinated brown rice on number of aberrant crypt foci (ACF) after 8 weeks of treatment**. The data were analyzed by using one way ANOVA and the values are expressed as mean ± S.E.M. *p *< 0.05 was considered significant.

There was a significant difference (*p *< 0.05) in the body weight between the groups treated with GBR and the control (G1) (data not shown). The effect of GBR on the incidence of ACF is shown in Table 1 and Figure 1. It indicates that there was a significant reduction (*p *< 0.05) in the total number of ACF and AC, and multicrypt of ACF per colon in all the groups treated with GBR (G2, G3 and G4) compared to the control group (G1). Reduction in the total number of ACF and AC, and multicrypt of ACF with increase in the percentage of GBR was observed but yet insignificant.

**Table 1:** Effect of germinated brown rice on AOM-induced ACF formation in male *Sprague Dawley *rats

		**No. of ACF containing**				
			
**Treatment**	**ACF/colon**	**1 crypt**	**2 crypts**	**3 crypts**	**4 or more crypts**	**Total aberrant crypt**
		
**AOM + basal diet** **(G1)**	41.50 ± 3.008	16.00 ± 0.730	15.67 ± 1.520	4.00 ± 0.894	5.50 ± 1.310	81.30 ± 7.745
**AOM + basal diet + 2.5 g/kg of GBR** **(G2)**	28.60 ± 1.720^a^	12.20 ± 1.241	10.60 ± 1.208^a^	2.20 ± 0.200	3.40 ± 0.678	53.60 ± 3.970^a^
**AOM + basal diet + 5 g/kg of GBR** **(G3)**	25.50 ± 2.327^a^	12.00 ± 2.160	9.50 ± 0.646^a^	2.75 ± 0.250	1.25 ± 0.479^a^	44.25 ± 2.750^a^
**AOM + basal diet +10 g/kg of GBR** **(G4)**	24.40 ± 1.806^a^	10.40 ± 1.123^a^	8.40 ± 0.927^a^	2.20 ± 0.374	2.80 ± 0.860	44.00 ± 4.159^a^
**Basal diet alone (G5)**	0	0	0	0	0	0

Values are expressed as mean ± SEM. Mean with the ^a ^superscript are significant *p *< 0.05 as compared to positive control, G1

**Figure 2:**
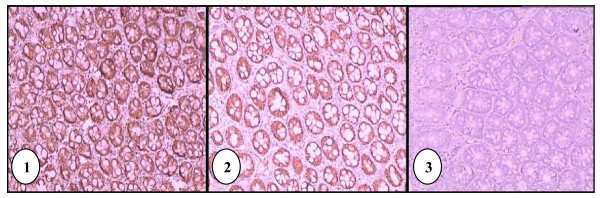
**Immunohistochemical staining of positive control (G1) tissue using antibody against β-catenin**. Intense immunoreactivity was present throughout the epithelial cells, in cell membrane and cytoplasm (100× magnification) **(1)**. Immunohistochemical staining of G2 tissue using antibody against β-catenin showing less intense staining (100× magnification) **(2)**. Immunohistochemical staining of negative control (G5) tissue using antibody against β-catenin. (100× magnification)**(3)**.

All tissue samples from G1 showed intense and heterogenous β-catenin immunoreactivity throughout the colon epithelia (Figure 2). In samples from the treatment groups (G2, G3 and G4), heterogenous expression of β-catenin was also observed. None of the tissue samples from G5 showed expression of β-catenin. There was no localization of β-catenin at cell membrane, cytoplasm and nucleus of the colon epithelial cells. The median scores of β-catenin expression and mean ranks by Kruskal-Wallis test are shown in Table 2. Comparison between groups by Mann-Whitney U-test showed significant difference (*p *= 0.0001) in the expression of β-catenin.

**Table 2:** Median scores and mean ranks of β-catenin expression according to group

Group	Median score*	Mean score
**AOM + basal diet** **(G1)**	6.00^a^	300.83
**AOM + basal diet + 2.5 g/kg of GBR** **(G2)**	5.00^b^	223.68
**AOM + basal diet + 5 g/kg of GBR** **(G3)**	5.00^b^	222.94
**AOM + basal diet +10 g/kg of GBR** **(G4)**	4.00^c^	184.94
**Basal diet alone (G5)**	0.00^d^	39.50

*Median scores with different superscript are significantly different at p < 0.05 (Mann-Whitney U-test).

**Figure 3:**
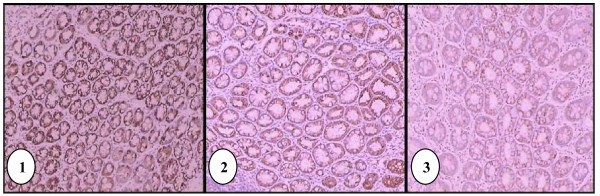
**Immunohistochemical staining of positive control (G1) tissue using antibody against COX-2**. Immunoreactivity was present throughout the epithelial cells (100× magnification) **(1)**. Immunohistochemical staining of G2 tissue using antibody against COX-2 showing weaker staining compared to G1 (100× magnification) **(2)**. Immunohistochemical staining of negative control (G5) tissue using antibody against COX-2 (100× magnification)**(3)**.

The colon epithelia from G1 showed more intense immunoreactivity of COX-2 indicating significant increase (*p *= 0.0001) in the expression of COX-2 compared to the treatment groups (G2, G3 and G4). Weak, diffused and heterogenous expression of COX-2 was observed in the tissue samples from G5 (Figure 3). Localization of COX-2 could be observed at nuclear membrane and cytoplasm of some of the colon epithelial cells. The median scores of COX-2 expression and mean ranks are shown in Table 3. Comparison between groups by Mann-Whitney U-test showed significant difference (*p *= 0.0001) in the expression of COX-2.

**Table 3:** Median scores and mean ranks of COX-2 expression according to group

Group	Median score*	Mean score
**AOM + basal diet** **(G1)**	6.00^a^	324.16
**AOM + basal diet + 2.5 g/kg of GBR** **(G2)**	5.00^b^	241.72
**AOM + basal diet + 5 g/kg of GBR** **(G3)**	4.00^c^	182.25
**AOM + basal diet +10 g/kg of GBR** **(G4)**	4.00^c^	170.04
**Basal diet alone (G5)**	3.00^d^	54.15

*Median scores with different superscript are significantly different at *p *< 0.05 (Mann-Whitney U-test).

Spearman rank correlation test showed significant positive linear relationship between total β-catenin and COX-2 score (Spearman's rho = 0.616, *p *= 0.0001).

## Discussion

This study demonstrates that treatment with 2.5 g/kg, 5 g/kg and 10 g/kg of GBR (weight of GBR (g)/weight of rat (kg)) significantly suppressed the total number of ACF, AC and multicrypt of ACF per colon, yet was insignificant among the GBR-treated groups. It is postulated that the chemopreventive effects of GBR was dose-independent. The ACF was found to consist of 1, 2, 3, 4 or more crypts. It is believed that the number of crypts increases with time due to crypt multiplication or branching [7]. The crypts reproduce themselves by a fission mechanism [29]. However, the number of ACFs correlates poorly with cancer risk. It is the crypt multiplicity that is more predictive of malignant transformation [30]. It has been hypothesized that many ACFs regress and only larger foci or lesions progress to cancer [9]. Therefore, GBR seems to be a potential agent to inhibit the progression of colon cancer since the number of larger foci (4 or more crypts) was found to be significantly less in all groups treated with it compared to the control group (Figure 1).

All tissue samples from G5, which represent the normal colon tissue did not show β-catenin immunoreactivity (Figure 2). There was no localization of stain at cell membrane, cytoplasm and nucleus of epithelial cells and goblet cells. This may indicate that the level of β-catenin in the normal colon mucosa is too low to be detected by this method of immunohistochemistry (IHC). In contrast, previous studies by Hong [31] reported that all the normal adjacent tissue of human colorectal sample showed β-catenin immunoreactivity in the plasma membrane at cell to cell border, and cytoplasm of the epithelial cells and goblet cells, but no nuclear staining was found. Takahashi *et al*. [32] also showed localization of β-catenin at membranes of cell-to-cell borders in normal colon epithelial cells of male F344 rats. The divergence in our staining result may be caused by inter-species and inter-strain variation between the *Sprague Dawley *rats and the other samples (human and other breeds of rats).

In the tissues of positive control group (G1), where colon carcinogenesis was initiated by injection of AOM but no treatment with GBR was given, intense β-catenin stain (immunoreactivity in the cytoplasm of epithelial cells is prominent) was observed, representing much higher level of β-catenin (Figure 2). The intensity of the brown stain is variable, where some cells exhibit greater intensity at the cell membrane compared to the cytoplasm, while other cells lost or have reduced brown stain at the cell membrane, thus appearing that the cytoplasm compartment has more localization of β-catenin. Overall scoring showed that the epithelial cells of G1, which was shown to have the highest number of aberrant crypt foci (ACF) among all groups (Table 1), has the highest score in the evaluation of immunohistochemical staining of β-catenin compared to other groups.

According to Goss and Groden [33], β-catenin binds to the cytoplasmic tail of α-catenin and E-cadherin and indirectly to the cytoskeleton. It may dissociate from them and enters cytoplasm as free unbound β-catenin. Since β-catenin is an important Wnt signaling pathway activator, its level in the normal tissue is constantly regulated by APC, axin and glycogen synthase kinase-3β (GSK-3β) complex that targets its rapid degradation in the cytoplasm to prevent translocation of β-catenin into the nucleus to promote transcription of various target genes [31].

Mutations in β *-catenin, APC, axin *and *GSK-3β *may result in the inability of the complex to phosphorylate β-catenin, resulting in failure of ubiquitination; whereas *E-cadherin *mutation can cause dissociation of β-catenin from the complex, releasing free β-catenin into the cytoplasm. This explains loss or reduced β-catenin protein at the cell-to-cell borders but an over-expression in the cytoplasm of the G1 tissues.

In the treatment groups (G2, G3, and G4), heterogenous expression of β-catenin was observed in the cell membrane and cytoplasm compartment. Nevertheless, all the three treatment groups showed significant decrease in β-catenin expression compared to G1. This indicates that GBR has the potential of reducing the expression of β-catenin. It is postulated that GBR is a natural chemopreventive agent which acts through pathway involving β-catenin.

Tissue samples from G5, which represent the normal colon tissue showed some degree of COX-2 immunoreactivity, where the expression of COX-2 was found to be weak, diffused and heterogenous (Figure 3). Lack of expression of COX-2 in tissue samples from G5 indicates that most normal colonic cells were not undergoing inflammation. However there was still a score of 3 as to indicate that inflammation does exist in normal condition but at a very slow rate. COX-2 is known to be an inducible enzyme that plays an important role in induction of pain and inflammation [34]. Localization of COX-2 could be seen mainly at the cytoplasm of colon epithelial cells, and some cells also showed clear nuclear membrane stain. No nuclear staining was observed. Similarly, Tomozawa *et al*. [35] and Takahashi *et al*. [36] have also reported COX-2 expression in normal rat colon mucosa using IHC method.

In the tissues of G1, the colon epithelia showed much greater COX-2 immunoreactivity compared to all other groups (Figure 3). The staining was also heterogenous throughout the tissues, mostly in the cytoplasm. No nuclear staining was found. Many studies reported well-documented observation of COX-2 expression in colonic tumour. For example, Tomozawa *et al*. [35] and Takahashi *et al*. [36] have reported positive immunoreactivity in cytoplasm and nuclear membranes of tumour cells. The staining pattern in the treatment groups is similar, but with apparent reduction in both intensity and percentage of positive cell, compared to G1. This shows that GBR prevents colon carcinogenesis by down-regulating the COX-2 expression.

As for the comparison among the treatment groups, there were significant differences (*p *= 0.0001) between G2 and G3, and between G2 and G4. This indicates that the expression of COX-2 decreased with increase in the concentration of GBR. *APC, β-catenin, axin*, and *GSK-3β *mutations can cause increased expression of β-catenin in the cytoplasm and nucleus of colorectal carcinoma, which leads to Wnt pathway activation. Since COX-2 may be a downstream target of Wnt signaling pathway, *COX-2 *gene is upregulated and COX-2 expression is increased in tumour cells [37,38].

Spearman rank correlation test showed a statistically positive linear relationship between β-catenin and COX-2 scores. This is in accordance with findings by Kawasaki *et al*. [39] that showed positive correlation of COX-2 over expression with cytoplasmic β-catenin expression. Thus, indicating a possible link between COX-2 and the Wnt signaling pathway [40]. COX-2 has been shown to activate β-catenin through prostaglandin E2 and G protein-coupled receptor EP2 (E prostanoid 2) [41]. A study by Lee and Jeong has shown that β-catenin stabilizes COX-2 mRNA by interacting with AU-rich elements in a 3' untranslated region [42]. These findings suggest the existence of a positive feedback loop between β-catenin and COX-2. Therefore, GBR might have suppressed β-catenin expression through regulation of COX-2 or vice versa. It is also possible that GBR regulates expression of both proteins simultaneously.

This study indicates that GBR possesses good chemopreventive effects, more likely due to the nutritional components that are increasing in content following the process of germination such as phytic acid (IP6), ferulic acid, inositol and dietary fiber [4]. It has been reported that inositol hexaphosphate (IP6) reduced the carcinogen-induced large bowel cancer and inhibited growth of transplanted tumors [43]. However, the mechanism by which IP6 exerts chemopreventive is not completely understood. In general, IP6 is rapidly absorbed by cells and metabolized to lower phosphates and inositol [44]. Both IP6 and its lower phosphates have metal chelating activity and may interfere with tumor formation by suppressing metal catalyzed oxidation of fats. It regulates the cell cycle to block uncontrolled cell division and force malignant cells either to differentiate or to undergo apoptosis [45]. Alternatively, IP6 may block the activity of key enzyme(s) affecting cell proliferation. One enzyme candidate is PI-3kinase which plays a central role in signal transduction and cell transformation triggered by growth factor or tumor promoter. IP6 has been reported to inhibit PI-3 kinase activity *in vitro *[46]. In addition, the antioxidant properties of IP6 may also contribute to its antineoplastic activity [47]. Ferulic acid inhibits the growth of colonic ACF and suppresses the progression of preneoplastic to malignant neoplasia. The suppression of metabolic activation and enhancement of detoxification may become one of the mechanisms for chemopreventive action of ferulic acid [48].

As functional food component, Shamsuddin [44] has reported that inositol plays a role in suppressing tumor formation. Meanwhile, Alabaster [49] has found that the dietary fiber is linked to the prevention of colon carcinogenesis. A high fiber intake result in a high stool bulk, which reduces stool transmit time and thus lowers exposure to potential carcinogens [50].

This findings show that germinated brown rice has chemopreventive properties in rats induced with colon cancer. The administration of 2.5 g/kg, 5 g/kg and 10 g/kg of GBR (weight of GBR (g)/weight of rat (kg)) significantly reduced the total number of ACF, AC, and multicrypt of ACF, and the expression of β-catenin and COX-2 compared to the positive control (without GBR). It is suggested that GBR can be a promising dietary intake for prevention of human colon cancer.

## Competing interests

The authors declare that they have no competing interests.

## Authors' contributions

SYL was the chief investigator for this project in planning the study design and contributed to manuscript preparation. NA, HTT and YA took part in planning the study design, scoring of ACF and AC, immunohistochemistry analysis of β-catenin and Cox-2, statistical analysis and manuscript preparation. AHN processed the GBR. SN and IH assisted in scoring of ACF and AC, and immunohistochemistry analysis of β-catenin and Cox-2. MS performed the statistical analysis. IM was responsible for funding, study design and GBR supply. All authors read and approved the final manuscript.
